# Long non-coding RNA CASC9 promotes tumor progression in oral squamous cell carcinoma by regulating microRNA-545-3p/laminin subunit gamma 2

**DOI:** 10.1080/21655979.2021.1977103

**Published:** 2021-10-06

**Authors:** Baoshan Ning, Songya Guo, Yine Mei

**Affiliations:** Department of Stomatology, Wuhan Dongxihu District People’s Hospital, Wuhan, Hubei, China

**Keywords:** Oral squamous cell carcinoma, lncRNA CASC9, miR-545-3p, LAMC2

## Abstract

Long non-coding RNA (lncRNA) CASC9 is reported to be a tumor promoter in oral cancer, but its mechanism in oral squamous cell carcinoma (OSCC) has not been fully explored. Our study aimed to identify the interaction between lncRNA CASC9, microRNA-545-3p (miR-545-3p), and laminin subunit gamma 2 (LAMC2) in OSCC cells. Our study confirmed that lncRNA CASC9 and LAMC2 were upregulated in OSCC, whereas miR-545-3p expression was reduced. After performing a series of cell functional experiments, it was found that knockdown of lncRNA CASC9 or LAMC2 resulted in the inhibition of proliferation, colony formation, and migration of OSCC cells, but their negative effects could be partly impaired by the miR-545-3p inhibitor. In addition, we proved for the first time that lncRNA CASC9 can sponge miR-545-3p to upregulate LAMC2. In conclusion, our study revealed that lncRNA CASC9 promotes the malignancy of OSCC cells by sponging miR-545-3p to enhance LAMC2 expression, implying that lncRNA CASC9/miR-545-3p/LAMC2 may be an intervention approach in OSCC therapy.

## Introduction

Oral squamous cell carcinoma (OSCC), which accounts for 80% of all head and neck carcinomas, is a common malignant tumor worldwide [1,2]. At present, OSCC therapy and diagnosis have advanced, but the 5-year survival rate of patients with OSCC is still low (50%) [3]. OSCC is often diagnosed as other oral lesions, resulting in delayed treatment [4]. Therefore, a complete understanding of the molecular mechanism of OSCC progression is crucial for identifying novel therapeutic targets and diagnostic markers for OSCC.

Long non-coding RNAs (lncRNAs) with a length of >200 nucleotides (nt) are dysregulated in cancer [5–7]. Accumulating evidence has shown that lncRNAs act as key regulators in OSCC by controlling cellular physiological processes involving microRNA (miRNA) sponging, which regulates the target gene of miRNA [8,9]. For example, lncRNA SNHG20 facilitates the malignancy of OSCC by sponging miR-19b-3p to upregulate RAB14 [10]. lncRNA CASC9 is overexpressed in many human cancers, including gastric [11], esophageal squamous cell [12], and breast cancers [13]. lncRNA CASC9 is a tumor promoter in oral cancer [14,15], but its regulatory mechanism involving the downstream key miRNA/mRNA axis is still unclear.

miRNAs, 22 nucleotides in length, are a large group of small non-coding RNAs that can exert crucial biological functions by binding to the three prime untranslated region (3ʹUTR) of their target genes [16,17]. Recently, many miRNAs have been shown to participate in OSCC progression by targeting the genes, including miR-579 [18], miR-487a-3p [19], and miR-210-3p [15]. miR-545-3p, a member of the miRNA family, exerts an inhibitory function in multiple cancers [20–22]. For example, miR-545-3p, sponged by lncRNA PTPRG-AS1, inhibits cell tumorigenicity in ovarian cancer by attacking its target gene, HDAC4 [23]. However, the function of miR-545-3p in OSCC has not been investigated.

Bioinformatics analysis includes the Gene Expression Omnibus (GEO) database, which is often used to predict key genes in OSCC [24]. In our study, bioinformatics analysis predicted that lncRNA CASC9 might promote OSCC development by regulating the miR-545-3p/ laminin subunit gamma 2 (LAMC2) axis. LAMC2 together with laminin subunit beta-3 (LAMB3) and laminin subunit alpha-3 (LAMA3) constitutes laminin-332, which is secreted by epithelial tumor cells, thereby regulating cancer invasion [25]. Chen et al. [26] showed that LAMC2 was differentially expressed in 119 patients with OSCC using Affymetrix U133 microarray. Zhou et al. [27] revealed that LAMC2 targeted by miR-134 could accelerate migration and invasion in OSCC. However, the regulatory mechanism of LAMC2 on lncRNA CASC9 and miR-545-3p in OSCC has not been elucidated.

Together with the findings of the previous studies, we suspected that lncRNA CASC9, miR-545-3p, and LAMC2 might exert important functions in OSCC. Therefore, the aim of our study was to reveal the interaction and function of lncRNA CASC9, miR-545-3p, and LAMC2 in OSCC, which may provide novel insights into therapeutic targets and diagnostic markers for OSCC.

## Materials and methods

### Bioinformatics analysis

GEPIA (http://gepia.cancer-pku.cn/index.html) was used to show the expression of lncRNA CASC9 in cancer samples. GSE37991, obtained from another database (GEO DataSets, https://www.ncbi.nlm.nih.gov/gds/?term=), is an mRNA expression microarray, including OSCC samples and non-tumor samples. Therefore, in this study, GSE37991 was used to screen the upregulated genes in OSCC samples with adj.P < 0.05 and log_2_FC>2. Then, the GO enrichment of upregulated genes in OSCC was analyzed using STRING (https://string-db.org/) to identify the key gene (LAMC2). Next, TargetScan and starBase were used to predict the miRNAs that could target LAMC2, and starBase was used to predict the miRNAs sponged by lncRNA CASC9. Finally, the key miRNA (miR-545-3p) connecting lncRNA CASC9 and LAMC2 was overlapped from TargetScan and starBase using Venny 2.1.0.

## Clinical samples and cell culture

The paired tumor and adjacent normal samples (2 cm from the tumor) were collected from 32 patients with OSCC (age range, 46–71 years) at Wuhan Dongxihu District People’s Hospital between April 2019 and July 2020. Our study was performed in accordance with the Declaration of Helsinki and was approved by the Ethics Committee of Wuhan Dongxihu District People’s Hospital. The clinical characteristics of the patients with OSCC used in this study are shown in **Supplementary Table I**.

Human oral epithelial cells (HOECs; cat. no. BNCC340217) were purchased from BNCC (China), whereas SCC-4 (cat. no. CRL-1624), CAL-27 (cat. no. CRL-2095), and SCC-9 (cat. no. CRL-1629) were oral epithelial cells obtained from American Type Culture Collection (ATCC, USA). All cells were maintained in Dulbecco’s modification of Eagle’s medium (DMEM) and 10% fatal bovine serum (FBS) at 37°C and 5% CO_2_.

## Real-time quantitative polymerase chain reaction (RT-qPCR)

Total RNA was extracted from tissues or cells using the Total RNA Extraction Kit (cat. no. R1200, Solarbio, China). After calculating RNA concentration using an ultraviolet photometer (Bio-Rad, USA), the QuantiTect Reverse Transcription Kit (Qiagen, Germany) was used to synthesize cDNA for 1 μg RNA. The miScript SYBR Green PCR Kit (cat. no. 218,075, Qiagen, Germany) was used to perform RT-qPCR under the following reaction conditions: 95°C for 15 min, 40 cycles at 94°C for 15 s, 55°C for 30 s, and 70°C for 30 s). Finally, the relative expression of miRNA and mRNA was calculated using the 2^−ΔΔCt^ method with the internal references (U6 and GAPDH). The primer sequences purchased from RiboBio (China) are shown in **Supplementary Table II**.

## Cell transfection

Small interfering RNAs (siRNAs) for knockdown of lncRNA CASC9 (si-CASC9-1 and si-CASC9-2), LAMC2 (si-LAMC2), miR-545-3p mimic, miR-545-3p inhibitor, and their corresponding negative controls, including si-NC, mimic-NC, and inhibitor-NC, were obtained from Shanghai GenePharma (Shanghai, China). For transfection, SCC-4 and SCC-9 cells at 60% confluence were transfected with 50 nM siRNA for lncRNA CASC9 or LAMC2, miR-545-3p mimic/inhibitor, and their corresponding negative control using Lipofectamine 3000 (Invitrogen, USA). After 48-h incubation, the transfection efficiency was determined using RT-qPCR, and follow-up experiments were conducted. The vector sequences used in this study are shown in **Supplementary Table III**.

## Cell proliferation assay

Cell Counting Kit-8 (CCK-8, cat. no. C0037, Beyotime, China) was used to detect cell proliferation, according to a previous study [28]. Briefly, 6000 SCC-4 and SCC-9 cells were plated to 96-well plates and incubation overnight. After transfection for 0, 24, 48, and 72 h, 90 μl/well DMEM medium with 10 μl/well CCK-8 was added to cells and incubated for 2 h Finally, OD_450_ was detected by a microreader.

## Colony-formation assay

Colony-formation assay was performed according to the previous study [28]. After different transfections, SCC-4 and SCC-9 cells (200 cells/well) were seeded in 6-well plates for colony-formation assay. After culturing the cells for 10 days at 37°C, the colonies were fixed using methanol for 30 min at 25°C and stained with 0.5% crystal violet (cat. no. C0775; Sigma-Aldrich; USA) for 20 min at 25°C. Finally, the images of colonies were photographed using an inverted microscope.

## Cell migration assay

Transwell assay (Corning, USA) was used to detect cell migration according to a previous study [29]. Briefly, 500 μL serum-free DMEM medium containing 5 × 10^5^ transfected cells was added to the upper chamber, and 450 μL DMEM medium and 50 μL FBS were added to the lower chamber. After 24 h, methanol was used to fix the cells that migrated to the lower chamber for 30 min at 25°C, and the cells were stained with 0.5% crystal violet for 20 min at 25°C. Finally, images of cell migration were obtained using an inverted microscope.

## Identification of targeting relationship

A luciferase assay was performed to confirm the targeting relationship between lncRNA CASC9, miR-545-3p, and LAMC2, according to a previous study [30]. The psiCHECK-2 vectors, including wild-type (WT) lncRNA CASC9 or LAMC2 3ʹ-untranslated regions (3ʹUTR) with the binding sites and mutant (MUT) lncRNA CASC9 or LAMC2 without the binding sites were purchased from Shanghai GenePharma (Shanghai, China). Then, the mimic-NC or miR-545-3p mimic was co-transfected with psiCHECK-2-CASC9-WT/MUT or psiCHECK-2-LAMC2-WT/MUT to SCC-4 and SCC-9 cells using Lipofectamine 3000. After 48 h, the dual-luciferase reporter assay system (cat. no. E1910, Promega, USA) was used to measure the luciferase activity of firefly and Renilla cells.

## RNA immunoprecipitation (RIP) assay

The RIP assay was performed in SCC-4 and SCC-9 cells with 48 h transfection of miR-545-3p mimic or mimic-NC using Magna RIP RNA Binding Protein Immunoprecipitation Kit (Millipore) according to a previous study [31]. The transfected cells were lysed in RNA lysis buffer, and then the magnetic beads conjugated to anti-Argonaute 2 (Ago2) antibody or mouse immunoglobulin G (IgG) were added to the lysate. After proteinase K treatment, the precipitated RNA was collected to detect the enrichment of lncRNA CASC9 using RT-qPCR.

## Western blotting

Western blotting was performed as previously described [29]. Total protein was extracted using radioimmunoprecipitation assay buffer (cat. no. 20–188, Sigma; USA). After separating 20 µg total protein using 12% SDS-PAGE, the protein was transferred to PVDF membranes for blocking for 3 h using 5% nonfat milk. Next, the blocked membranes were washed with TBST and reacted with primary antibodies, including LAMC2 (cat. no. ab274376, Abcam, USA) and GAPDH (cat. no. ab9485, Abcam, USA), overnight at 4°C. Subsequently, the membranes were incubated with HRP-linked rabbit antibodies (1:5000, cat. no. ab6721, Abcam, USA) for 1 h at 25°C. The membranes were incubated with SuperEnhanced chemiluminescence detection reagent (Applygen, China), and the protein blot was covered with plastic wrap to expose the X-ray film.

## Statistical analysis

GraphPad 8.0 (GraphPad Software, Inc., USA) was used for statistical analysis with paired Student’s t-test for two groups and one-way or two-way ANOVA with Dunnett’s or Tukey’s post hoc test for multiple groups. The correlation between lncRNA CASC9, miR-545-3p, and LAMC2 was analyzed using Pearson’s correlation analysis. All the data from three independent experiments are presented as mean ± standard deviation, and p-values less than 0.05, were considered to indicate significant differences.

## Results

In our study, we predicted that the lncRNA CASC9/miR-545-3p/LAMC2 axis may play a key role in OSCC by bioinformatics analysis. Therefore, we aimed to investigate the biological role of the lncRNA CASC9/miR-545-3p/LAMC2 axis in OSCC. Our data showed that lncRNA CASC9 and LAMC2 were upregulated in OSCC, and silencing lncRNA CASC9 or LAMC2 suppressed the malignant phenotype of OSCC cells. Moreover, miR-545-3p/LAMC2 axis was the downstream of lncRNA CASC9 and partly reversed the effect of lncRNA CASC9 and LAMC2 on OSCC cells. In summary, our findings revealed the function of the lncRNA CASC9/miR-545-3p/LAMC2 axis in OSCC cells, which may provide a novel approach for OSCC therapy.

## miR-545-3p/LAMC2 may be the downstream target of lncRNA CASC9 in OSCC

**Figure 1.**
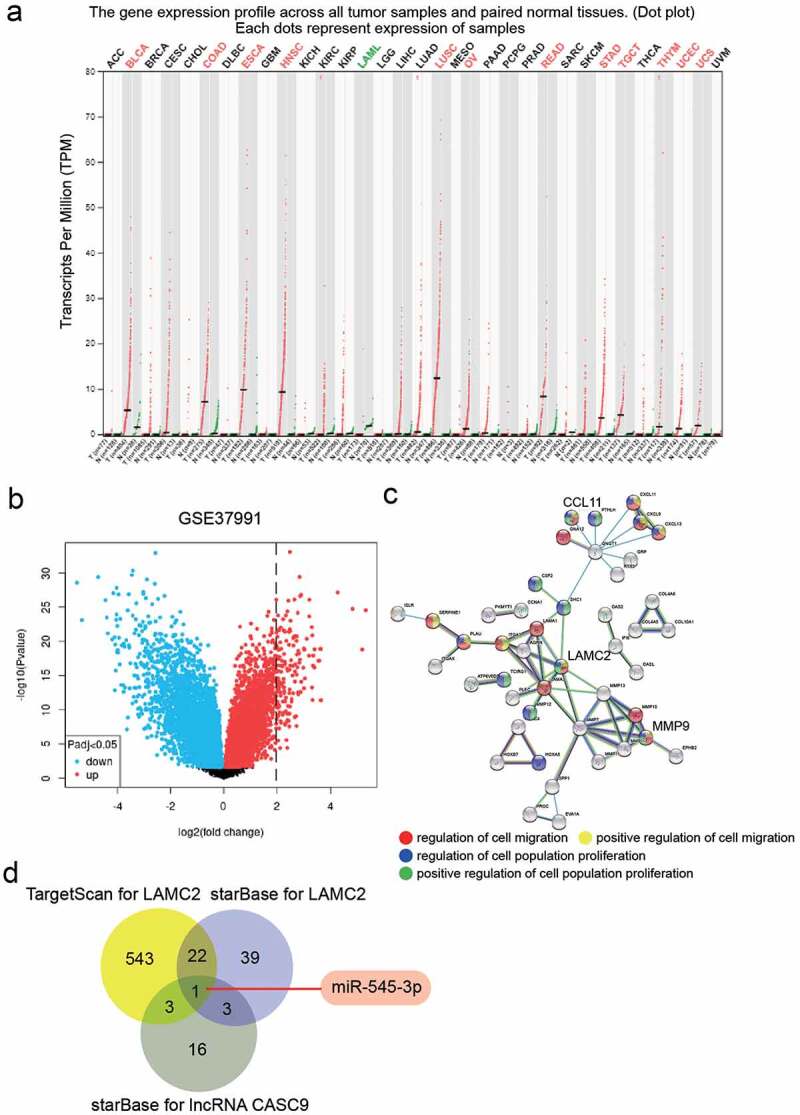
lncRNA CASC9 might play the key role in OSCC by miR-545-3p/LAMC2. (a) The expression of lncRNA CASC9 in multiple cancer types based on GEPIA database. Red color represents the CASC9 expression in tumor samples, and green color represents the CASC9 expression in normal samples. (b) The 130 upregulated genes in OSCC samples from a mRNA microarray GSE37991 was identified by limma 3.26.8 with the filter criteria of adj.P < 0.05 and log2FC>2. (c) Three key genes were identified to be associated with cell migration and cell proliferation by STRING GO enrichment. (d) miR-545-3p was confirmed as the common miRNA targeting LAMC2 and lncRNA CASC9 by the prediction of TargetScan and starBase

From the GEPIA database, lncRNA CASC9 was upregulated in multiple cancer types (Figure 1a). As for OSCC, lncRNA CASC9 promotes tumor progression, but its downstream targeting of the miRNA/mRNA axis is not fully understood. GSE37991 from the GEO database was used to identify key mRNAs (Figure 1b). With the filter criteria of adj.P < 0.05, and log_2_FC>2, 130 upregulated genes were selected by limma 3.26.8. Uploading the upregulated genes to STRING for GO enrichment, the results showed that CCL11, LAMC2, and MMP9 were associated with cell migration and cell proliferation, which are closely related to tumor progression (Figure 1c). LAMC2 is the central factor that connects most genes, and its effect has not been explored in OSCC; hence, LAMC2 has attracted our attention. StarBase and TargetScan were used to predict the miRNAs targeting LAMC2, whereas starBase was used to predict the miRNAs sponged by lncRNA CASC9. Finally, miR-545-3p was identified as a miRNA that could target lncRNA CASC9 and LAMC2 (Figure 1d).

## Effect of lncRNA CASC9 on OSCC cells

**Figure 2.**
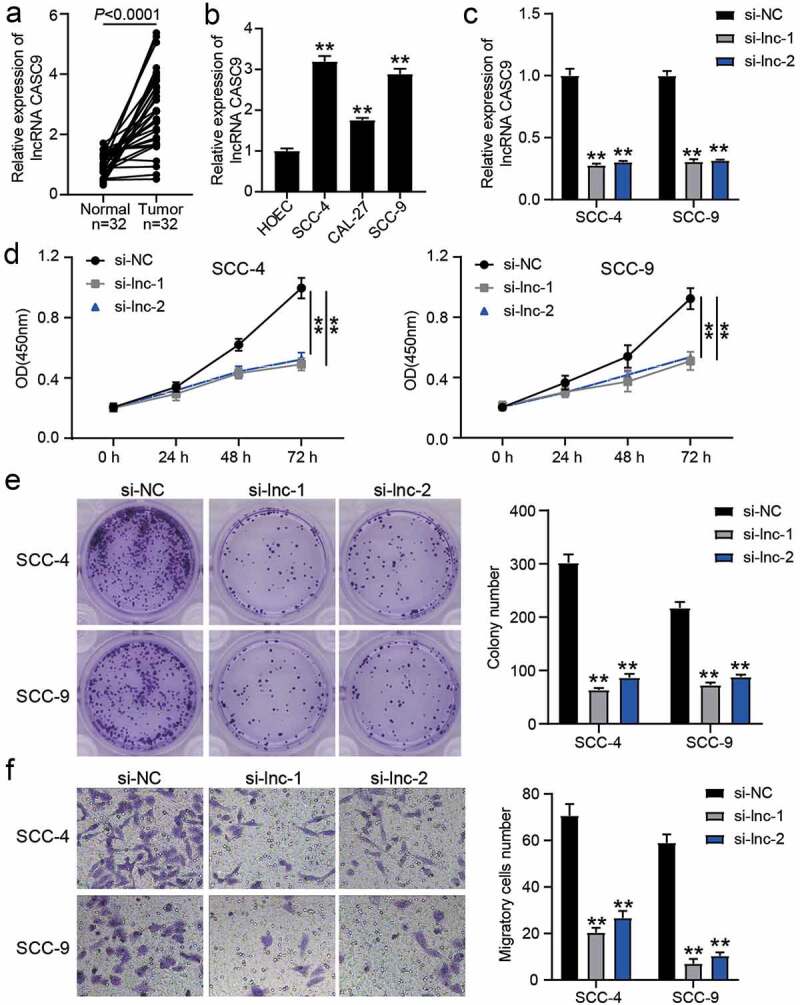
The effect of si-CASC9 in OSCC cells. (a) The expression of lncRNA CASC9 in OSCC tissues and paired adjacent tissues from 32 OSCC patients. The data were analyzed by with paired student’s t-test. (b) The expression of lncRNA CASC9 in OSCC cells (SCC-4, CAL-27 and SCC-9) and human oral epithelial cells (HOEC). **P < 0.01 compared with HOEC using one-way ANOVA. (c) The expression of lncRNA CASC9 in SCC-4 and SCC-9 cells with the transfection of si-CASC9 was identified by RT-qPCR. (d) The cell proliferation in SCC-4 and SCC-9 cells with the transfection of si-CASC9 was detected by CCK8 assay. (e) The colony formation in SCC-4 and SCC-9 cells with the transfection of si-CASC9 was measured by colony formation assay. (f) The cell migration in SCC-4 and SCC-9 cells with the transfection of si-CASC9 was assessed by transwell assay. (c-f) **P < 0.01 compared with si-NC using two-way ANOVA. si-NC, siRNA negative control. si-lnc-1 and si-lnc-2, two siRNAs for CASC9

To verify the effect of lncRNA CASC9 in OSCC, the expression of lncRNA CASC9 in OSCC tissues and cell lines was verified by RT-qPCR, which showed that lncRNA CASC9 expression was elevated in OSCC tissues and cells compared to that in normal adjacent tissues or HOECs (Figure 2a and 2b). Owing to the higher expression of lncRNA CASC9 in SCC-4 and SCC-9 cells, si-CASC9 was transfected into these two cell lines (Figure 2c). CCK8 assay showed that silencing CASC9 suppressed cell proliferation (Figure 2d), and colony-formation assay showed that silencing CASC9 suppressed colony formation capability (Figure 2e). Moreover, the ability of cell migration was also impaired in SCC-4 and SCC-9 cells transfected with si-CASC9 (figure 2f).

## lncRNA CASC9 could sponge miR-545-3p

**Figure 3.**
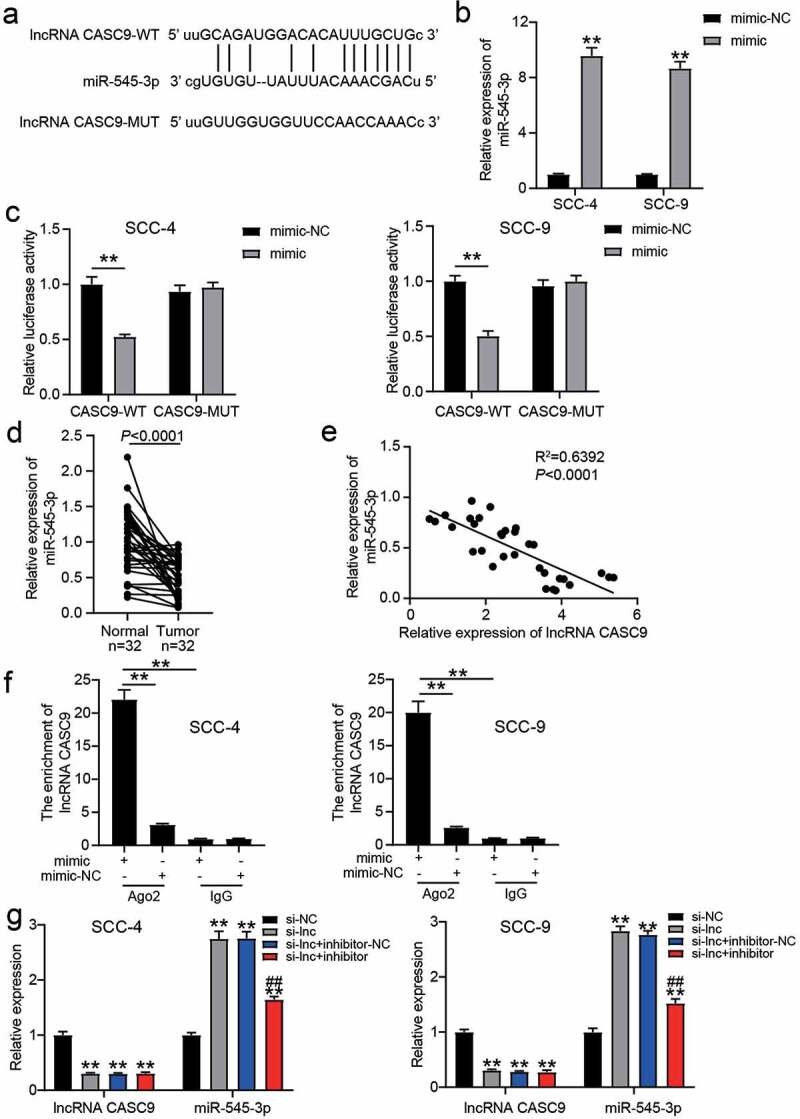
miR-545-3p was the downstream of lncRNA CASC9. (a) The binding sites between lncRNA CASC9 and miR-545-3p by starBase prediction. (b) The expression of miR-545-3p in SCC-4 and SCC-9 cells with the transfection of miR-545-3p mimic was confirmed by RT-qPCR. **P < 0.01 compared with mimic-NC using two-way ANOVA. (c) The luciferase activity was detected by luciferase assay in SCC-4 and SCC-9 cells with the co-transfection of CASC9-WT/CASC9-MUT and miR-545-3p mimic/mimic-NC. **P < 0.01 using two-way ANOVA. (d) The expression of miR-545-3p in OSCC tissues and paired adjacent tissues from 32 OSCC patients. The data were analyzed by paired student’s t-test. (e) The correlation between miR-545-3p and lncRNA CASC9 in OSCC tissues was analyzed by Pearson’s correlation analysis. (f) The enrichment of lncRNA CASC9 in miR-545-3p mimic group or mimic-NC group was detected by RIP assay. **P < 0.01 using two-way ANOVA. (g) The expression of lncRNA CASC9 and miR-545-3p in SCC-4 and SCC-9 cells with the transfection of si-CASC9 and/or miR-545-3p inhibitor. **P < 0.01 compared with si-NC. ##P < 0.01 compared with si-lnc+inhibitor-NC. The data were analyzed by two-way ANOVA. WT, wild-type. MUT, mutant. mimic, miR-545-3p mimic. si-NC, siRNA negative control. si-lnc, si-CASC9. inhibitor-NC, inhibitor negative control. inhibitor, miR-545-3p inhibitor

The wild-type (WT) lncRNA CASC9 with the binding sites for miR-545-3p and the mutant (MUT) lncRNA CASC9 without the binding sites for miR-545-3p are shown in Figure 3a. After miR-545-3p mimic successfully transfected SCC-4 and SCC-9 cells (Figure 3b), the luciferase assay showed that the luciferase activity in co-transfection of lncRNA CASC9-WT and miR-545-3p mimic group was reduced by approximately 50%, whereas the other groups did not show significant changes (Figure 3c). In clinical samples, it was found that miR-545-3p expression reduced in OSCC tissues (Figure 3d), and its expression was negatively correlated with lncRNA CASC9 expression in OSCC tissues (Figure 3e). The RIP assay further confirmed that the enrichment of CASC9 was observed in the miR-545-3p mimic group in the presence of Ago2 (figure 3f). After transfecting miR-545-3p inhibitor, it was found that miR-545-3p inhibitor did not affect the decrease in lncRNA CASC9 expression caused by si-CASC9, but si-CASC9 enhanced miR-545-3p expression (Figure 3g).

## miR-545-3p inhibitor regulated the effect of si-CASC9 on OSCC cells

**Figure 4.**
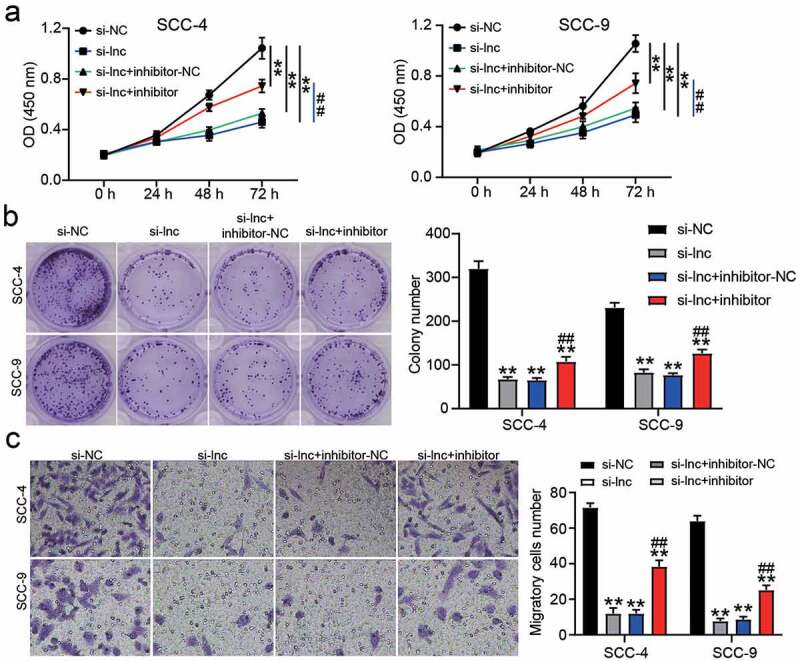
miR-545-3p inhibitor affect the function of si-CASC9 in OSCC cells. (a) The cell proliferation in SCC-4 and SCC-9 cells with the transfection of si-CASC9 and/or miR-545-3p was detected by CCK8 assay. (b) The colony formation in SCC-4 and SCC-9 cells with the transfection of si-CASC9 and/or miR-545-3p inhibitor was measured by colony formation assay. (c) The cell migration in SCC-4 and SCC-9 cells with the transfection of si-CASC9 and/or miR-545-3p inhibitor was assessed by transwell assay. **P < 0.01 compared with si-NC. ##P < 0.01 compared with si-lnc+inhibitor-NC. The data were analyzed by two-way ANOVA. si-NC, siRNA negative control. si-lnc, si-CASC9. inhibitor-NC, inhibitor negative control. inhibitor, miR-545-3p inhibitor

To confirm the regulatory effect of miR-545-3p on OSCC cells, we performed a series of cell functional experiments. The CCK8 assay showed that inhibiting miR-545-3p could relieve the inhibitory effect of si-CASC9 on the proliferation of OSCC cells (Figure 4a). Similar to cell proliferation, miR-545-3p knockdown enhanced the colony formation capability compared to that in the si-CASC9 group (Figure 4b), and the number of migratory cells increased after co-transfection of si-CASC9 and miR-545-3p inhibitor groups compared to that in the si-CASC9 group (Figure 4c).

## LAMC2 was the target of miR-545-3p

**Figure 5.**
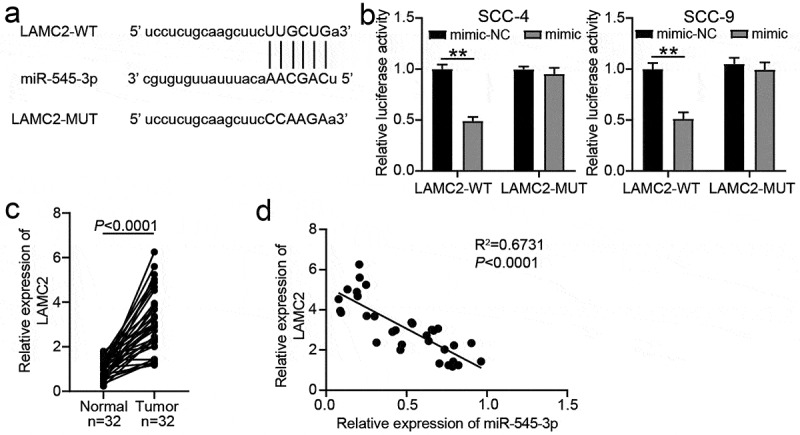
LAMC2 was the target of miR-545-3p. (a) The binding sites between LAMC2 and miR-545-3p by starBase prediction. (b) The luciferase activity was detected by luciferase assay in SCC-4 and SCC-9 cells with the co-transfection of LAMC2-WT/LAMC2-MUT and miR-545-3p mimic/mimic-NC. **P < 0.01 using two-way ANOVA. (c) The expression of LAMC2 in OSCC tissues and paired adjacent tissues from 32 OSCC patients. The data were analyzed by paired student’s t-test. (d). The correlation between miR-545-3p and LAMC2 in OSCC tissues was analyzed by Pearson’s correlation analysis. WT, wild-type. MUT, mutant. mimic, miR-545-3p mimic

The WT LAMC2 3ʹUTR with binding sites and MUT LAMC2 3ʹUTR without the binding sites are shown in Figure 5a. After the luciferase assay, it was found that the miR-545-3p mimic reduced the luciferase activity in the WT LAMC2 3ʹUTR group, whereas it did not influence the activity in the MUT LAMC2 3ʹUTR group (Figure 5b). In the clinical samples, LAMC2 expression was higher in the OSCC samples than in the normal adjacent samples (Figure 5c), and LAMC2 expression was negatively correlated with miR-545-3p expression (Figure 5d).

## Downregulation of miR-545-3p regulated the effect of LAMC2 knockdown on OSCC cells

**Figure 6.**
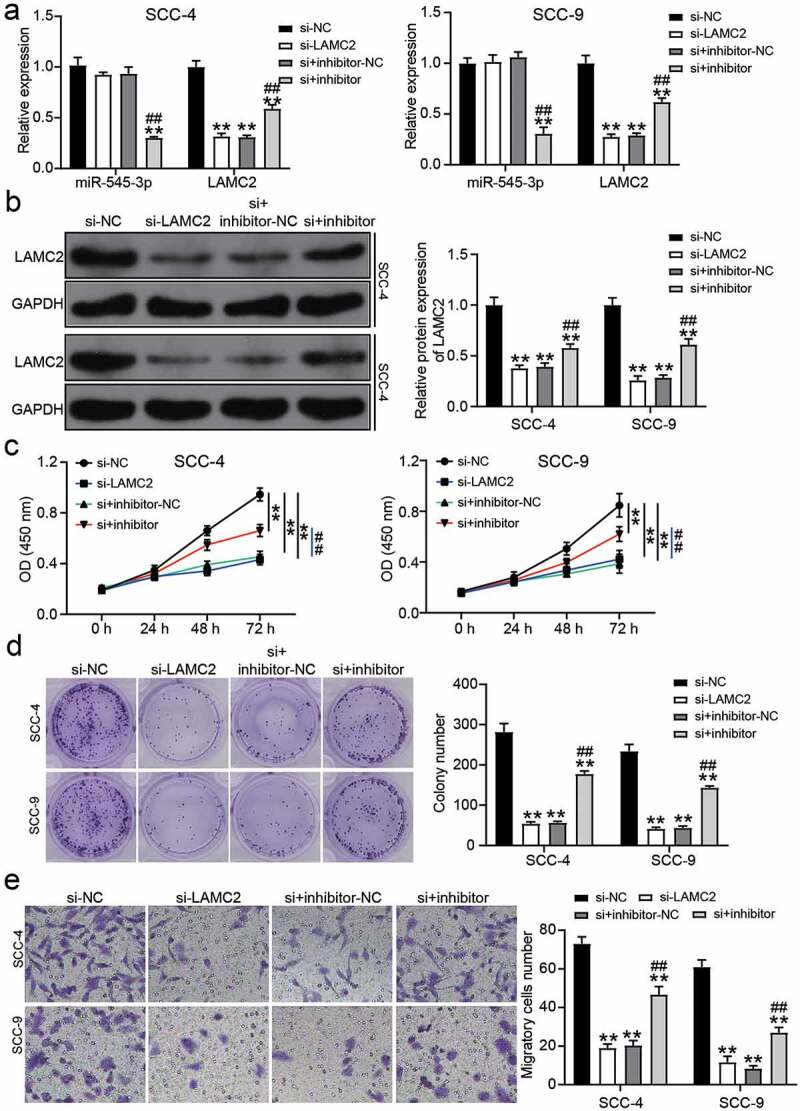
miR-545-3p inhibitor affect the function of si-LAMC2 in OSCC cells. (a) The expression of miR-545-3p and LAMC2 in SCC-4 and SCC-9 cells with the transfection of si-LAMC2 and/or miR-545-3p inhibitor was detected by RT-qPCR. (b) he expression of miR-545-3p and LAMC2 in SCC-4 and SCC-9 cells with the transfection of si-LAMC2 and/or miR-545-3p inhibitor was detected by western blotting. (c) The cell proliferation in SCC-4 and SCC-9 cells with the transfection of si-LAMC2 and/or miR-545-3p was detected by CCK8 assay. (d) The colony formation in SCC-4 and SCC-9 cells with the transfection of si-LAMC2 and/or miR-545-3p inhibitor was measured by colony formation assay. (e) The cell migration in SCC-4 and SCC-9 cells with the transfection of si-LAMC2 and/or miR-545-3p inhibitor was assessed by transwell assay. *P < 0.05, **P < 0.01 compared with si-NC. ##P < 0.01 compared with si-lnc+inhibitor-NC. The data were analyzed by two-way ANOVA. si-NC, siRNA negative control. inhibitor-NC, inhibitor negative control. inhibitor, miR-545-3p inhibitor

Based on RT-qPCR and western blot analysis, si-LAMC2 reduced LAMC2 expression, but miR-545-3p inhibitor relieved the downregulation of LAMC2 caused by si-LAMC2 in OSCC cells (Figure 6a and 6b). For cell proliferation, si-LAMC2 suppressed cell proliferation, but this inhibitory effect was relieved by co-transfection with miR-545-3p inhibitor (Figure 6c). Colony-formation assay showed that si-LAMC2 played an inhibitory role in colony formation, and the miR-545-3p inhibitor partly neutralized this inhibitory effect (Figure 6d). Cell migration assay showed a similar result to colony formation, suggesting that LAMC2 knockdown reduced the number of migratory cells, whereas co-transfection of si-LAMC2 and miR-545-3p inhibitor elevated the number of migratory cells compared to that in the si-LAMC2 group (Figure 6e).

## Discussion

lncRNAs are considered key biomarkers for cancer diagnosis and therapeutic targets for cancer treatment [32,33]. Several studies have reported that lncRNAs play a key role in OSCC progression, such as lncRNA HOXA11-AS [34], lncRNA UCA1 [35], and lncRNA JPX [8]. In our study, we found that lncRNA CASC9 was upregulated in OSCC, and lncRNA CASC9 knockdown attenuated proliferation, colony formation, and migration of OSCC cells. Meanwhile, we proved that miR-545-3p sponged by lncRNA CASC9 was downregulated in OSCC, and it could relieve the effect of lncRNA CASC9 on OSCC cells. We also found that silencing LAMC2, which was targeted by miR-545-3p, could inhibit the malignancy of OSCC cells. Taken together, we showed that lncRNA CASC9 contributes to the malignancy of OSCC cells by sponging miR-545-3p to upregulate LAMC2.

Recently, several studies have found that lncRNA CASC9 is overexpressed in cancers, and it plays oncogenic roles in human cancer types, including colorectal and gastric cancers [36,37]. For instance, lncRNA CASC9 accelerates cell proliferation and cell cycle progression in breast cancer by binding to the miR-194/497 cluster to regulate checkpoint kinase 1 [13]. Yang et al. demonstrated that lncRNA CASC9 expression increased in OSCC, and its upregulation was associated with clinical stage and overall survival time in patients with OSCC [14]. Moreover, their team uncovered that lncRNA CASC9 could change the activity of the AKT/mTOR pathway to promote OSCC progression. Similar to the previous study on lncRNA CASC9 in OSCC, our study further confirmed that lncRNA CASC9 has an oncogenic influence on OSCC cells by regulating proliferation, colony formation, and migration. However, we revealed that lncRNA CASC9 regulates the progression of OSCC cells by sponging miR-545-3p to upregulate LAMC2, a mechanism different from that reported by Yang et al. Therefore, the regulatory mechanism of lncRNA CASC9 in OSCC has expanded.

miR-545-3p, a member of the miRNA family, has been proven to be a tumor suppressor in multiple cancers, such as lung cancer and ovarian cancer [21,23]. Specifically, in a previous report, miR-545-3p sponged by lncRNA PTPRG-AS1 to target histone deacetylase 4 suppressed cell migration and invasion in epithelial ovarian cancer [23]. However, the function of miR-545-3p in OSCC has not yet been investigated. Our study fills this gap in the literature. Here, bioinformatics analysis and luciferase assay confirmed that miR-545-3p was downregulated in OSCC tissues and was the downstream target of lncRNA CASC9 in OSCC cells, suggesting that miR-545-3p might be a key miRNA in OSCC. By performing a series of cell functional assays, we proved that miR-545-3p inhibitor could relieve the inhibitory effect of silencing lncRNA CASC9 on cell proliferation, colony formation, and cell migration in OSCC cells by targeting LAMC2. Our findings indicate that miR-545-3p is a tumor suppressor in OSCC, which is consistent with the findings of the previous studies on the inhibitory function of miR-545-3p in other cancer types.

An increasing number of studies have confirmed that LAMC2 expression is upregulated in human cancers, including lung adenocarcinoma and ovarian cancer [30,38]. For instance, LAMC2 is overexpressed and targeted by miR-125a-5p in ovarian cancer, and its high expression promotes the progression of ovarian cancer via the p38-MAPK signaling pathway [30]. In OSCC, Chen et al. found the upregulation of LAMC2 in 119 patients with OSCC using Affymetrix U133 2.0 Plus arrays, but they did not further explore the function of LAMC2 in OSCC. In 2020, Zhou et al. revealed that LAMC2 targeted by miR-134 contributed to tumor stem cell migration and invasion in OSCC [27]. Similar to the findings of the previous studies on LAMC2 in OSCC, our study revealed that silencing LAMC2 impedes proliferation, colony formation, and migration of OSCC cells. However, in contrast to a previous study, we confirmed that LAMC2 was downstream target of the lncRNA CASC9/miR-545-3p axis, and its effect on OSCC cells could be partly reduced by miR-545-3p. In addition, a positive correlation between LAMC2 and lncRNA CASC9 was demonstrated, which was consistent with the study published in 2018, showing that lncRNA CASC9 and LAMC2 have positive correlation in esophageal squamous cell carcinoma by interacting with CREB-binding protein [12]. In contrast to a previous study, we proved for the first time that miR-545-3p is the bridge connecting the lncRNA CASC9 and LAMC2 in OSCC.

This study revealed the regulatory mechanism of the lncRNA CASC9/miR-545-3p/LAMC2 axis in OSCC cells. However, our study has several limitations. First, LAMC2 was shown to regulate cancer progression by activating the p38-MAPK signaling pathway [30] or PI3K-Akt signaling pathway [27]. Therefore, the downstream signaling pathway of the lncRNA CASC9/miR-545-3p/LAMC2 axis in OSCC involving signaling pathways needs to be further explored in the future. In addition, we demonstrated the function of lncRNA CASC9/miR-545-3p/LAMC2 axis *in vitro*. However, its function should be deeply investigated *in vivo* and in clinical settings. Our future study will focus on the role of the lncRNA CASC9/miR-545-3p/LAMC2 axis *in vivo*.

## Conclusion

In conclusion, our current study revealed that lncRNA CASC9 and LAMC2 were overexpressed and miR-545-3p was downregulated in OSCC. Moreover, lncRNA CASC9 can contribute to cell proliferation, colony formation, and cell migration in OSCC cells by sponging miR-545-3p to upregulate LAMC2 expression. Our findings provide a possible therapeutic approach to OSCC therapy.

## Supplementary Material

Supplemental Material

## Data Availability

All data generated or analyzed during this study are included in this published article.

## Highlights

1. lncRNA CASC9 promotes the malignancy of OSCC cells by sponging miR-545-3p/LAMC2 axis.

2. Downregulation of LAMC2 attenuates the malignancy of OSCC cells.

3. miR-545-3p can partly relieve the effect of lncRNA CASC9 or LAMC2 on OSCC cells
