# Supplementary material for: Long non-coding RNA CASC9 promotes tumor progression in oral squamous cell carcinoma by regulating microRNA-545-3p/laminin subunit gamma 2
Source: Bioengineered. 2021 Oct 6;12(1):7907–19. doi: 10.1080/21655979.2021.1977103 (PMC8806561; doi:10.1080/21655979.2021.1977103)
Supplement: Supplemental Material [file KBIE_A_1977103_SM2239.zip › supplementary/Supplementary table I revised.docx]

**Supplementary table I** The clinical characteristics of 32 OSCC patients in this study

| **Characteristics** | **N=32** |
| --- | --- |
|  |  |
| **Age (years)** |  |
| ≤60 | 17 |
| >60 | 15 |
| **Gender** |  |
| Male | 16 |
| Female | 16 |
| **T classification** |  |
| T1 and T2 | 12 |
| T3 and T4 | 20 |
| **N classification** |  |
| N0 | 14 |
| N+ | 18 |
| **Tumor differentiation** |  |
| Well | 8 |
| Moderate | 6 |
| Poor | 18 |
