# Supplementary material for: Long non-coding RNA CASC9 promotes tumor progression in oral squamous cell carcinoma by regulating microRNA-545-3p/laminin subunit gamma 2
Source: Bioengineered. 2021 Oct 6;12(1):7907–19. doi: 10.1080/21655979.2021.1977103 (PMC8806561; doi:10.1080/21655979.2021.1977103)
Supplement: Supplemental Material [file KBIE_A_1977103_SM2239.zip › supplementary/Supplementary table II revised.docx]

**Supplementary table II** The sequences of the primers in this study

| **Primer** | **Sequences** |
| --- | --- |
| **lncRNA CASC9** | Forward: 5'- TGGTCAGCCACATTCATGGT-3' |
|  | Reverse: 5'-AGTGCCAATGACTCTCCAGC-3' |
| **miR-545-3p** | Forward: 5'-TGGCTCAGTTCAGCAGGAAC-3' |
|  | Reverse: 5'-CTCAACTGGTGTCGTGGA-3' |
| **LAMC2** | Forward: 5'-TACAGAGCTGGAAGGCAGGATG-3' |
|  | Reverse: 5'-GTTCTCTTGGCTCCTCACCTTG-3' |
| **GAPDH** | Forward: 5'-CAGCCTCAAGATCATCAGCA-3' |
|  | Reverse: 5'-GGCATGGACTGTGGTCATGAG-3' |
| **U6** | Forward: 5'-CTCGCTTCGGCACA-3' |
|  | Reverse: 5'-AACGCTTCACGAATTTGCGT-3' |
