# Supplementary material for: Long non-coding RNA CASC9 promotes tumor progression in oral squamous cell carcinoma by regulating microRNA-545-3p/laminin subunit gamma 2
Source: Bioengineered. 2021 Oct 6;12(1):7907–19. doi: 10.1080/21655979.2021.1977103 (PMC8806561; doi:10.1080/21655979.2021.1977103)
Supplement: Supplemental Material [file KBIE_A_1977103_SM2239.zip › supplementary/Supplementary table III revised.docx]

**Supplementary table III** The sequences of vectors in this study

| **Primer** | **Sequences** |
| --- | --- |
| **si-CASC9-1** | Forward: 5'-GGGCAUUGAGAAGUUAGAATT-3' |
|  | Reverse: 5'-UUCUAACUUCUCAAUGCCCTT-3' |
| **si-CASC9-2** | Forward: 5'-GGACUCAUAUUACCAGUCUTT-3' |
|  | Reverse: 5'-AGACUGGUAAUAUGAGUCCTT-3' |
| **si-LAMC2** | Forward: 5'-GCAGGUUGAGACUACUUAA-3' |
|  | Reverse: 5'-UUAAGUAGUCUCAACCUGC-3' |
| **miR-545-3p mimic** | 5'-UCAGCAAACAUUUAUUGUGUGC-3' |
| **miR-545-3p inhibitor** | 5'-GCACACAAUAAAUGUUUGCUGA-3' |
| **si-NC** | 5'-UUCUCCGAACGUGUCACGUTT-3' |
| **Mimic-NC** | 5'-UUCUCCGAACGUGUCACGUTT-3' |
| **Inhibitor-NC** | 5'-CAGUACUUUUGUGUAGUACAA-3' |
